# Immediate Effects of Ipsilateral Forearm-Palm Sliding Stimulation on Glove-Type Numbness After Surgery for Cervical Spondylotic Myelopathy: A Case Report

**DOI:** 10.7759/cureus.103057

**Published:** 2026-02-05

**Authors:** Shinya Iki, Tomoya Ishigaki, Youki Teraoka, Takuya Kawaguchi

**Affiliations:** 1 Department of Rehabilitation, Kawaguchi Neurosurgery Rehabilitation Clinic, Hirakata City, JPN; 2 Department of Physical Therapy, Faculty of Health Sciences, Kio University, Koryo, JPN; 3 Department of Occupational Therapy, Graduate School of Health Sciences, Yamagata Prefectural University of Health Sciences, Yamagata, JPN; 4 Department of Neurosurgery, Kawaguchi Neurosurgery Rehabilitation Clinic, Hirakata City, JPN

**Keywords:** numbness, physical therapy, postoperative cervical spondylotic myelopathy, sensory disturbance, tactile stimulation

## Abstract

This report aims to investigate the possibility of a new physical therapy intervention for postoperative numbness after cervical myelopathy. The patient was a male in his early 80s. He started outpatient rehabilitation 18 days after laminoplasty, posterior fusion, and intervertebral foramen widening surgery. He began outpatient rehabilitation on the 18th day after vertebroplasty and posterior fusion, and on the 18th day after intervertebral foramen magnification. From 108 days after surgery until the end of outpatient rehabilitation on postoperative day 151, a therapist applied simultaneous sliding tactile stimulation to two points, namely, the ipsilateral palm and forearm, moving gently toward the distal direction. The results showed immediate improvement in numbness, superficial sensation, pain perception, and fine motor control. Simultaneous contact stimulation of the palm with numbness and the ipsilateral forearm without numbness may be a new intervention method to immediately improve numbness and tactile sensation after cervical spondylotic myelopathy surgery and to contribute to the improvement of fine motor control.

## Introduction

Cervical spondylotic myelopathy (CSM) is a condition characterized by sensory disturbances in the upper limbs and motor dysfunction in both the upper and lower limbs. CSM results from spinal cord compression caused by age-related canal stenosis and vertebral instability, such as degenerative changes in the intervertebral discs, posterior bulging, and vertebral osteophytes. While there have been several isolated reports on the clinical course of CSM, evidence regarding the effectiveness of rehabilitation aimed at improving residual symptoms after surgery is limited. Numbness involving the upper limb, in particular, often poses challenges in terms of physical therapy because of its negative impact on motor function and quality of life (QOL).

Inoue et al. [1] found that 48% of patients still had severe numbness at 12 months after surgery and that those with improvement in numbness at 12 months had better physical function and QOL. Therefore, residual numbness may negatively impact motor function and QOL.

Numbness is an umbrella term that can refer to sensory hypersensitivity, abnormal sensations, sensory dullness, and occasionally motor dysfunction, but is most commonly used to describe sensory hypersensitivity or abnormal sensations. In the International Classification of Diseases, 10th Edition, numbness is classified as a sensory disorder of the skin and includes sensory loss, illusions/perceptual abnormalities, abnormal sensations, and sensory dullness. Therefore, numbness encompasses a wide range of symptoms that are difficult to classify, making diagnosis and treatment challenging. It has been reported that numbness is difficult to improve with rehabilitation in patients with post-stroke pain [2]. Furthermore, the nature of the pain that occurs after a stroke (numbness, burning pain, cold hypersensitivity, throbbing pain, and pulsating pain) is similar to the symptoms after spinal cord injury, suggesting that numbness in CSM may follow a similar course [3].

In recent years, transcutaneous electrical stimulation has been found to be an effective treatment for disorders involving the spinal cord and central nervous system and may be a useful intervention for numbness [4]. However, there are practical limitations to its use in outpatient rehabilitation settings because of a lack of the necessary equipment and the time constraints associated with outpatient visits being greater than those associated with inpatient care. Therefore, alternative treatments for numbness are required.

In this report, we describe a case in which simultaneous sliding stimulation of two points on the same side of the forearm and palm resulted in an immediate reduction in numbness and changes in tactile sensation and dexterity in a patient who had undergone surgery for CSM and in whom physical therapy interventions for numbness had failed. This report also explores the potential of novel physical therapy interventions for numbness based on the known clinical course of CSM.

## Case presentation

**Figure 1:**
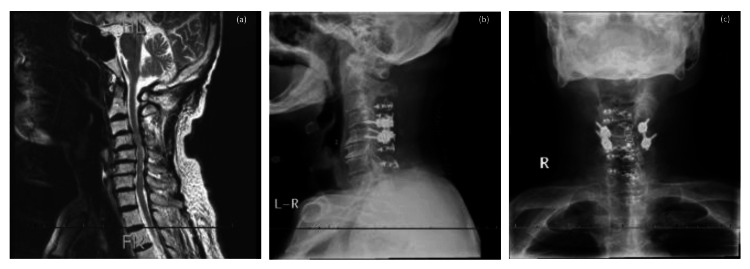
Findings on imaging before and after surgery. (a) Preoperative sagittal magnetic resonance image obtained 37 days before surgery showing cervical kyphosis with spinal canal stenosis, which was particularly evident at the C4/5 level. The patient subsequently underwent laminoplasty at C3-C7, posterior spinal fusion at C4/5, and foraminotomy at C5. (b) Sagittal and (c) frontal radiographs obtained on postoperative day 14 confirm screw insertion into the C4/5 pedicles and rod fixation.

The patient was a male in his early 80s with a history of type 2 diabetes, hypertension, surgery for colon and bladder cancer, and bilateral cataracts, and was receiving antidiabetic and antihypertensive medication. Approximately six months earlier, he had noticed persistent glove-shaped sensory disturbances and numbness in both upper limbs, along with muscle weakness in the right shoulder and elbow. This pattern of sensory impairment was consistent with glove-like numbness and sensory deficits typically observed when the C6 spinal segment is affected by compression at the C4/5 intervertebral level (Figure 1).

He underwent rehabilitation at an acute care hospital, but there was no subjective improvement in his sensory disturbances, numbness, or muscle weakness. He was discharged home on postoperative day (POD) 15. On POD 18, he started physical therapy in our outpatient rehabilitation department twice a week for 60 minutes per session. At this time, his body mass index was 23.5, and he was able to walk independently to the hospital. He was living independently but reported difficulties with tasks related to dressing, such as buttoning upper garments and zipping lower garments. His score on the Japanese Orthopaedic Association Cervical Myelopathy Evaluation Questionnaire (JOACMEQ) was 63/100, indicating moderate disability. His subscale scores were as follows: cervical spine function, 9/12; upper limb motor function, 5/10; lower limb motor function, 16/17; bladder function, 13/13; and QOL, 20/40. His symptom-related scores on the JOACMEQ [5] were 80 for neck and shoulder pain or stiffness, 0 for sensation of tightness in the chest, 90 for pain or numbness in the arms or hands, and 0 for pain or numbness from the chest to the feet (Table 1). At the start of outpatient rehabilitation, his JOACMEQ total score was 72, with the following subscale scores: cervical spine function, 9; upper limb motor function, 7; lower limb motor function, 16; bladder function, 13; and QOL, 27. His scores on a 100-mm visual analog scale (VAS) were 60 for neck and shoulder pain or stiffness, 0 for chest tightness, 85 for pain or numbness in the arms or hands, and 0 for pain or numbness from the chest to the feet. Although an improvement in upper limb function exceeding the minimal clinically important difference was observed, difficulties persisted with buttoning clothing because of sensory impairment and numbness (Table 1). Notably, during this period, glycosylated hemoglobin (HbA1c) levels, an indicator of diabetes control, remained at or below 6.0, and there were no changes in medication.

**Table 1:** Effect of ipsilateral forearm-palm sliding stimulation on physical and cognitive function and course of glove-type numbness during rehabilitation after surgery for cervical spondylotic myelopathy. 9HPT, Nine-Hole Peg Test; CSI, Central Sensitization Inventory Short Form test; JOACMEQ, Japanese Orthopaedic Association Cervical Myelopathy Evaluation Questionnaire; NRS, Numerical Rating Scale; POD, postoperative day; QOL, quality of life; VAS, visual analog scale.

Test			POD 18 (start of outpatient rehabilitation)	POD 92-106 (before the main physiotherapy intervention)	POD 108 (physical therapy intervention)	POD 151 (end of outpatient rehabilitation)	POD 241 (3 months after completion of outpatient rehabilitation)
JOACMEQ (score)	Total score		63	72	73	73	72
	Cervical spine function		9	9	9	9	8
	Upper limb motor function		5	7	8	8	7
	Lower limb motor function		16	16	15	15	16
	Bladder function		13	13	13	13	13
	QOL		20	27	28	28	28
	Neck and shoulder pain or stiffness, VAS (mm)		80	60	25	25	30
	Sensation of tightness in the chest, VAS (mm)		0	0	0	0	0
	Pain or numbness in the arms or hands, VAS (mm)		90	85	65	65	50
	Pain or numbness from the chest to the feet, VAS (mm)		0	0	0	0	0
9HPT (s)		Right	34	32	31	24	30
		Left	33	35	30	24	27
CSI (score)			-	2	-	-	-
Hand numbness, NRS score (0-10)		Right	7	7	5	5	7
		Left	9	9	8	8	8
Immediately after physical therapy intervention, hand numbness in the palm, NRS score (0-10)		Right	7	7	2	2	4
		Left	9	9	3	3	5

**Figure 2:**
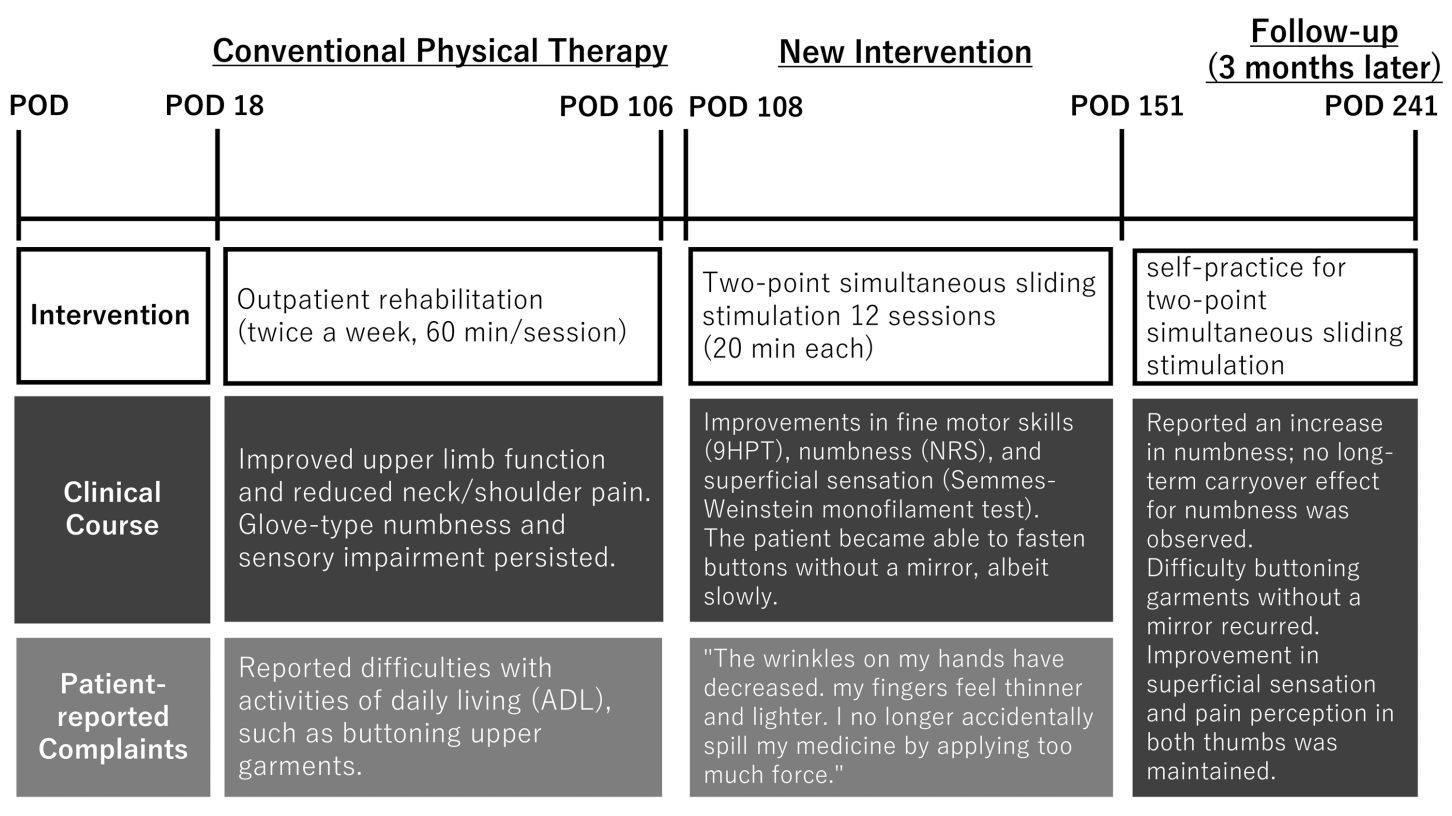
Timeline of physical therapy and the patient’s clinical course. POD, postoperative day.

The Nine-Hole Peg Test (9HPT) [6] was performed on POD 106 to evaluate finger dexterity and showed values of 31 seconds for the right hand and 30 seconds for the left hand; both scores were poorer than the average for the patient’s sex and age group (right hand, 25.8 seconds; left hand, 26.0 seconds) [7]. The Central Sensitization Inventory Short Form yielded a score of 2 out of 36, which was below the cutoff value of 20, indicating no symptoms related to central sensitization [8]. Numbness was rated as moderate to severe on the Numerical Rating Scale (NRS) [9], being 5/10 for the right hand and 8/10 for the left hand (Table 1). Superficial sensation was assessed using the Semmes-Weinstein monofilament test [10], with scores of 4.74 for both thumbs, 4.31 for the right index finger, 4.08 for the left index finger, 4.56 for both palms and dorsal surface of the hands, 3.61-3.84 for other fingers, and 3.84 for both the volar and dorsal aspects of both forearms. Assessment of temperature sensation using a thermal probe (Yufu Seiki Co., Ltd, Tokyo, Japan) based on the method described by Velstra et al. [11] revealed impaired sensation in both thumbs and both index fingers, with impairment of cold sensation in both thumbs and the left ring and little fingers. Pain perception was assessed using a quantitative perception needle (Yufu Seiki Co., Ltd) and found to be reduced in both thumbs at 12 g and in both index fingers at 4 g, while other fingers could perceive pain up to 1 g. Two-point discrimination was assessed using a Dellon 2 Point Disk-Criminator (Sakai Medical Co., Ltd., Tokyo, Japan), and values below the normal reference of 2 mm were noted for all fingers, both palms, and the dorsal surface of both hands. Overall, sensory impairment was observed mainly in the thumbs and index fingers (Table 2). General physical therapy, consisting of muscle strengthening and fine motor skills training, between POD 18 (the start of outpatient rehabilitation) and POD 106, improved the patient’s upper limb function and reduced his neck and shoulder pain, but the numbness and sensory impairment persisted. Drawing on the theory that pain arises from inconsistencies in sensory information in the central nervous system, we implemented sensory discrimination tasks [12] and rubber hand illusion tasks [13]. In the sensory discrimination tasks, which involve identifying three different surface materials, patients find it difficult initially to distinguish them but are usually able to do so after a single session. However, in our case, there was no improvement in numbness. Mirror therapy and the rubber hand illusion tasks similarly failed to yield satisfactory responses. The patient’s clinical course up to this point is shown in Figure 2.

**Table 2:** Effect of ipsilateral forearm-palm sliding stimulation on sensory function during rehabilitation after surgery for cervical spondylotic myelopathy. 〇, perceived; ×, not perceived; AF, anterior forearm; DF, dorsal surface; PS, palmar surface; PF, posterior forearm; POD, postoperative day.

		POD 92-106 (Before the main physiotherapy intervention)									POD 241 (3 months after completion of outpatient rehabilitation)								
		Thumb	Index	Middle	Ring	Little	PS	PF	AF	PF	Thumb	Index	Middle	Ring	Little	PS	PF	AF	PF
Semmes–Weinstein monofilament test (range, MIN-MAX = 1.65- 6.65)	Right	4.74	4.31	3.61	3.61	3.84	4.56	4.56	3.84	3.84	4.56	4.31	3.61	4.17	3.84	3.84	4.17	2.44	2.36
	Left	4.74	4.08	3.84	3.84	3.84	4.56	4.56	3.84	3.84	4.17	4.31	3.61	3.61	4.08	4.31	4.08	2.36	3.84
Thermal sensation (48±2℃)	Right	×	×	〇	〇	〇	〇	〇	〇	〇	〇	〇	〇	〇	〇	×	×	〇	〇
	Left	×	×	〇	〇	〇	〇	〇	〇	〇	×	×	×	×	×	×	×	〇	〇
Cold sensation (3±2℃)	Right	×	〇	〇	〇	〇	〇	〇	〇	×	×	〇	〇	〇	〇	×	×	〇	〇
	Left	×	〇	〇	×	×	〇	〇	〇	×	×	×	×	×	×	×	×	〇	〇
Pain perception (g) (range = 1-20 g)	Right	12	4	1	1	1	1	1	1	1	1	1	1	1	1	1	1	1	1
	Left	12	4	1	1	1	1	1	1	1	1	1	1	1	1	1	1	1	1
Two-point discrimination (mm)	Right	10	2	5	12	14	12	12	-	-	14	9	9	10	7	13	14	25	48
	Left	9	7	9	11	13	12	12	-	-	11	15	12	12	14	14	24	24	47

**Figure 3:**
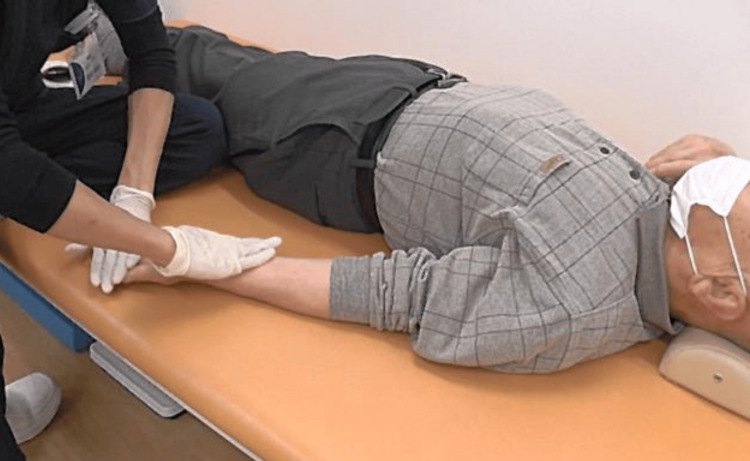
Physical therapy intervention session. The therapist used simultaneous contact stimulation by gently applying manual pressure toward the peripheral side at two points, namely, the anterior aspect of the forearm and the ipsilateral palm. This resulted in immediate improvement in fine motor skills, numbness, and superficial sensation.

On POD 108, based on conceptual insights gleaned from a previously reported method [14], we conceived an evaluation-oriented approach that addresses inconsistencies in sensory information on the affected side by referencing motor imagery on the non-affected side. Building on this concept, we introduced a novel intervention whereby simultaneous sliding tactile stimulation is applied to the ipsilateral limb to elicit immediate sensory responses, unlike in the previously reported approaches. Our patient presented with glove-like numbness and sensory impairment in both hands, making it difficult to obtain clear sensory input from the distal regions. We hypothesized that applying contact stimulation simultaneously at two points, namely, the anterior aspect of the forearm (a proximal site) and the distal ipsilateral palm surface, by gentle sliding of the therapist's hand toward the periphery while applying mild pressure, could promote cross-referencing and reorganization of sensory information within the central nervous system. This resulted in an immediate improvement in the 9HPT to 25 seconds on the right and 28 seconds on the left, with the NRS score for sensation of numbness in the hands improving to 1 for the right and 5 for the left. The Semmes-Weinstein monofilament test score was 4.56 for the right thumb and 4.08 for the left thumb, indicating improvement in numbness and superficial sensation (Figure 3).

**Figure 4:**
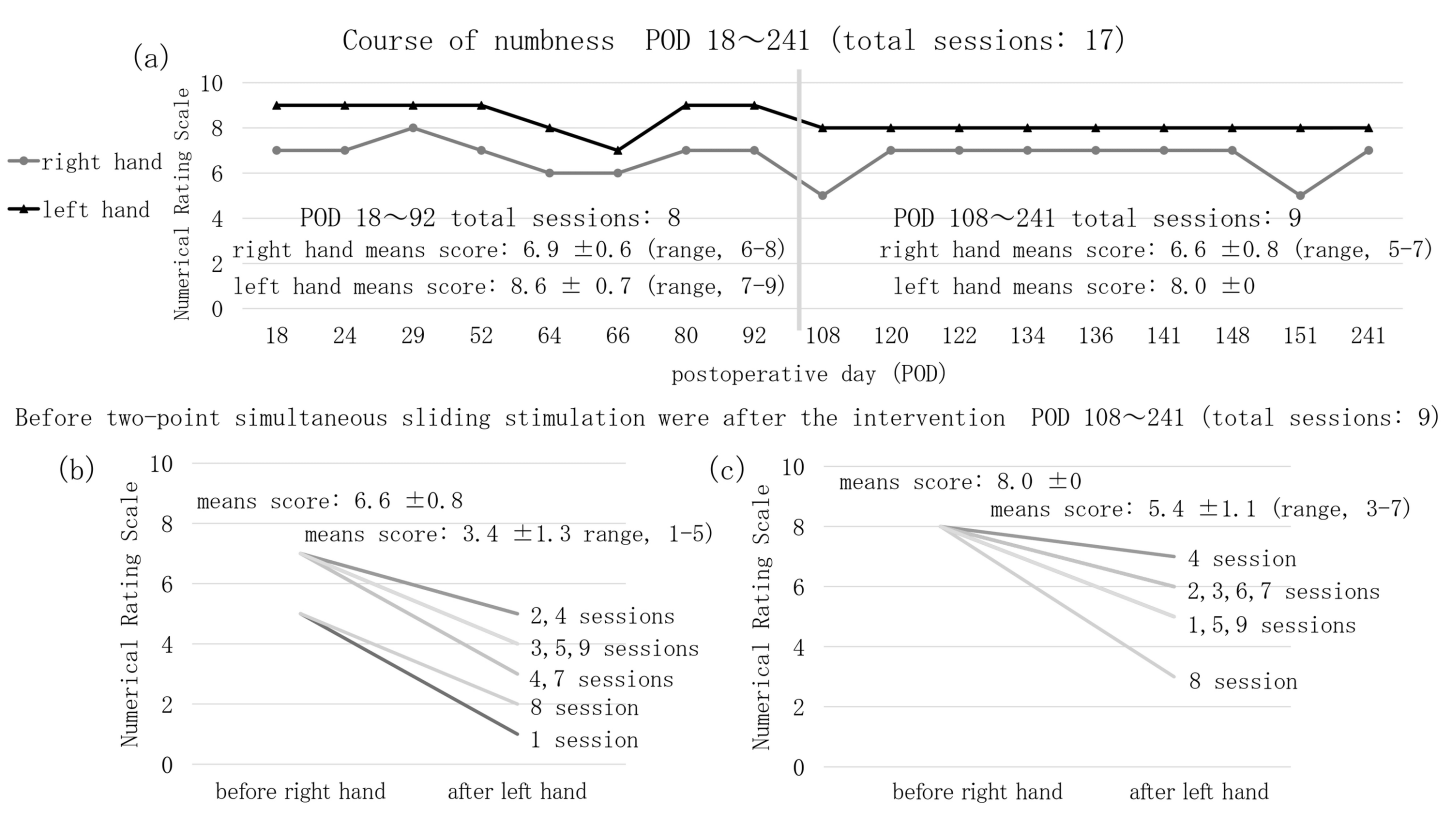
Course of numbness and immediate effect of two-point simultaneous sliding stimulation. (a) Numerical Rating Scale scores for numbness before the physical therapy intervention. The vertical line indicates the start of intervention using two-point simultaneous sliding stimulation. Unlike in the pre-intervention period (eight sessions between PODs 18 and 92), an effect was observed immediately after intervention with two-point simultaneous sliding stimulation (nine sessions between PODs 108 and 241). (b, c) Immediate effects before and after intervention with two-point simultaneous sliding stimulation (nine sessions in total), with improvement in numbness observed for both the right and left hands at each session. POD, postoperative day.

Therefore, 12 two-point simultaneous sliding stimulation sessions were performed, each lasting 20 minutes, with the patient in either the supine or seated position. Fine motor skills practice was continued until the end of outpatient rehabilitation on POD 151. The mean numbness scores on a 10-point NRS across eight sessions between POD 18 and POD 92 were 6.9 ± 0.6 (range, 6-8) for the right hand and 8.6 ± 0.7 (range, 7-9) for the left hand. The mean numbness scores across nine sessions between POD 108 and POD 241, before the two-point simultaneous sliding stimulation, were 6.6 ± 0.8 (range, 5-7) for the right hand and 8.0 ± 0.0 for the left hand. Following the intervention, the mean scores decreased to 3.4 ± 1.3 (range, 1-5) for the right hand and 5.4 ± 1.1 (range, 3-7) for the left hand. These findings indicated an immediate reduction in numbness, but with no observable carryover effect (Figure 4). This response was observed when the therapist's hands were applied, but not with soft materials, such as a sponge or a feather pillow.

**Figure 5:**
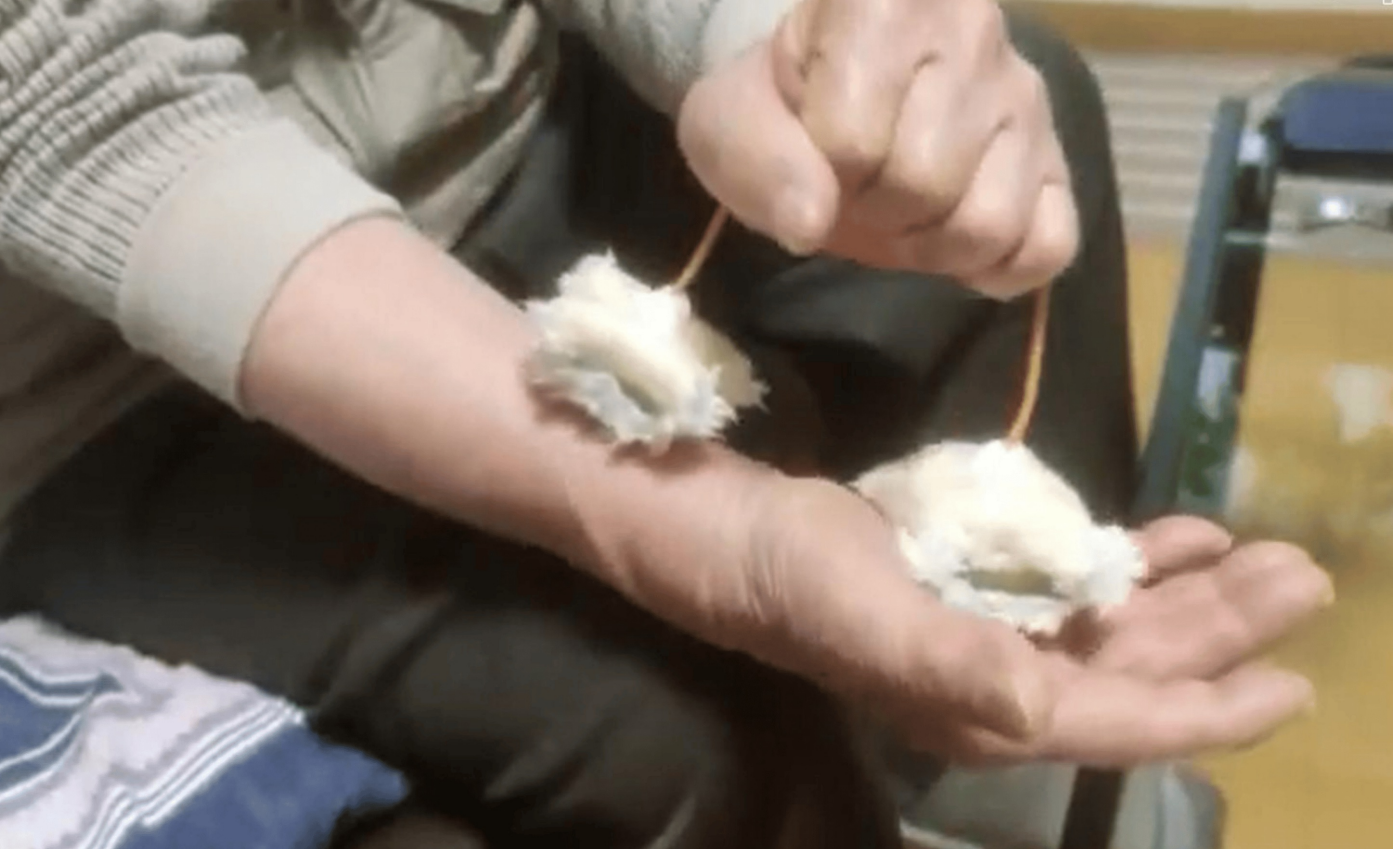
A self-practice tool for active self-touch and application of two-point simultaneous sliding stimulation. The tool was constructed by attaching wool, sponge, and wire to a low-temperature thermoplastic material, but this did not result in any significant reduction in numbness.

Furthermore, when contact stimulation was applied on the central side or at various segmental levels in the spine, there was no good response to stimulation of one point at C6 or another at C7 or to simultaneous stimulation of two points at C5/Th3 and C6/7 or at C6 and Th3. Slow sliding stimulation at a rate of approximately once per second yielded a good response. At the end of his outpatient rehabilitation session on POD 151, the patient was able to fasten the buttons of an upper garment without a mirror, albeit slowly. The patient reported improvements in sensory impairment, numbness, and his ability to perform activities of daily living, stating, “The wrinkles on my hands have decreased, and my complexion has improved. My fingers feel thinner and lighter. The numbness when washing dishes has improved. I no longer accidentally knock over medicine by applying too much force.” However, despite creating self-practice tools for active self-touch and two-point simultaneous sliding stimulation (Figure 5), no significant improvement was observed. During the follow-up period from POD 92 to POD 241 (three months after completion of outpatient rehabilitation), improvements were observed in the patient’s VAS scores for neck and shoulder pain and stiffness and in his JOACMEQ scores for numbness in the arms and hands (Table 1). However, while temporary improvements were noted in the 9HPT and NRS scores for numbness on POD 151 (when outpatient rehabilitation ended), the patient reported an increase in numbness three months later, by which time he found it difficult to button a garment without a mirror (Table 1). However, in terms of superficial sensation and pain perception, an improvement in both thumbs was observed on POD 241 (three months after completion of outpatient rehabilitation) (Table 2).

## Discussion

In this case, two-point simultaneous sliding stimulation of the ipsilateral forearm and palm based on neuroscientific insights resulted in an immediate reduction in numbness after surgery for CSM following the failure of general physical therapy and various interventions.

This effect was confirmed to be reproducible, suggesting involvement of a mechanism specific to this intervention. However, it should be noted that the outcomes described in this report were based on an isolated case, so caution is advised when considering the efficacy of this intervention and its neurophysiological mechanism of action.

The lack of efficacy of conventional physical therapy and various neuroscience-based interventions in this case may be attributed to their failure to correct sensory information mismatch. The patient may have relied on the sensation of numbness during sensory discrimination tasks, making it difficult to provide appropriate sensory input as a reference. Furthermore, the rubber hand illusion tasks did not promote integration between visual and bodily sensations. A possible explanation for this negative finding is that the patient also had numbness and superficial sensory dullness in the contralateral hand, which meant that the reference information necessary to induce the illusion was absent [15].

In this case, we applied two-point simultaneous sliding stimulation as an alternative physical therapy for numbness after surgery for CSM. The numbness was relieved immediately by simultaneous stimulation of the area with numbness and the area with preserved sensation on the same side. In healthy individuals, simultaneous application of infrared laser stimulation, which activates nociceptive free nerve endings derived from Aδ and C fibers in the epidermis, and tactile stimulation using a filament positioned on the dorsal side of the hand between the stimulation sites, suppressed the blink reflex, which is a marker of subcortical pain inhibition. Cortical responses to initial pain were also suppressed, and there was an increase in the pain threshold [16]. These responses were reported to occur not on the dorsal surface of the contralateral foot outside the somatic segment but on the ventral aspect of the ipsilateral forearm within the segment [17]. Applying these findings to our present case, two-point simultaneous sliding stimulation within the somatic segment may have inhibited ascending nociceptive input from Aδ and C fibers at the brainstem level, potentially blocking part of the nociceptive input characteristic of paresthesia. As a result, sensory information processing was modified at the cortical level, potentially leading to improvements in tactile sensation and fine motor skills.

Furthermore, the fact that sensory function in the forearm was relatively preserved in this case may have been an important factor in the success of this intervention. The C6 spinal segment was impaired by compression at the C4/5 vertebral level, with sensory deficits extending beyond the thumb and index finger in the C6 dermatome to the little finger in the C8 dermatome, presenting as glove-shaped numbness and a sensory deficit, and explained by damage to the posterior column of the spinal cord. These sensory deficits were observed in our case, and preservation of normal sensation in the forearm may have functioned as a modifying stimulus for the areas with numbness.

Our patient did not achieve independent self-practice, and no long-term effects of the intervention were observed. One possible explanation for the failure of the intervention in the long term is that the presence of numbness in both hands may have prevented the establishment of self-practice, given that self-contact did not provide comfortable tactile sensations [18]. High-quality tactile stimulation has been reported to contribute to pain relief. In this case, self-contact using the numb hand appeared to make it difficult for the patient to recall normal tactile sensations. Accordingly, tactile contact provided by another person under conditions less likely to induce numbness may have been more suitable. Notably, manual contact delivered by the therapist appeared to elicit a more pronounced response than contact using soft materials such as a sponge or feather pillow. However, while this interpretation is plausible, it remains speculative within the context of a single case report. Therefore, it is not possible to determine whether emotional contact with another human being directly inhibited the neural transmission associated with numbness or whether the observed responses reflected non-specific factors, such as psychological reassurance or expectation effects. Consequently, any association between therapist-delivered tactile contact and changes in sensory impairment or fine motor performance should be interpreted with caution. Schirmer et al. [19] reported that afferent transmission of C fibers was modulated by emotional contact, which is consistent with our findings in this case. However, it has been observed that there is an inverse U-shaped relationship between the speed of tactile stimulation and the pleasantness of contact, albeit with some individual differences [20]. With the accumulation of further cases, it will be possible to observe differences in responses to changes in speed when performing this intervention more objectively. For example, the possibility that tactile stimulation administered by the therapist instilled a sense of security and strong expectations of improvement, potentially modifying the numbness threshold as a placebo or Hawthorne effect, could not be ruled out in this case. Therefore, it was difficult to interpret the pure physical effects of this intervention separately from the psychological effects associated with the tactile stimulation provided by the therapist's hands. This is the primary limitation of this report.

Looking ahead, it will be necessary to address multiple clinical challenges, including the sustainability of self-practice, frequency of intervention, and the long-term reproducibility of tactile stimulation. While numbness is believed to arise from reduced inhibition of sensory information from both the central and peripheral nervous systems, the mechanisms involved remain unclear. While awaiting further research to elucidate the pathophysiological mechanisms, the potential of physical therapy interventions in the clinical setting should be explored further.

Despite the challenges, the findings in this case suggest that our simple, non-invasive manual technique, which does not require special equipment or electrical stimulation, may be associated with an immediate reduction in numbness. This technique has potential applications in outpatient or home-based rehabilitation settings where resources are limited.

## Conclusions

Application of two-point simultaneous sliding stimulation (i.e., stimulation applied to an area with abnormal sensory input causing numbness and an ipsilateral adjacent area with relatively preserved sensory input) by a therapist may evoke immediate responses in terms of glove-like numbness and tactile sensation after surgery for CSM, with accompanying changes in manual dexterity. These responses may partly reflect psychological expectation effects, and further investigation is needed to identify the intervention-specific elements underlying these observations.
